# Relationship between Physical Activity, Mediterranean Diet and Emotional Intelligence in Spanish Primary Education Students

**DOI:** 10.3390/children10101663

**Published:** 2023-10-07

**Authors:** Daniel Sanz-Martín, Félix Zurita-Ortega, Pilar Puertas-Molero, Rafael Caracuel-Cáliz, José Manuel Alonso-Vargas, Eduardo Melguizo-Ibáñez

**Affiliations:** 1 Faculty of Humanities and Social Sciences, Universidad Isabel I, 09003 Burgos, Spain; daniel.sanz6718@ui1.es; 2 Department of Didactics Musical, Plastic and Corporal Expression, Faculty of Education Science, University of Granada, 18071 Granada, Spain; pilarpuertas@correo.ugr.es (P.P.-M.); josemalonsov@correo.ugr.es (J.M.A.-V.); edumeliba@correo.ugr.es (E.M.-I.); 3 Department of Education, University of Almeria, 04120 Almeria, Spain; caracuel@ual.es; 4 Faculty of Education Science, Universidad Internacional de Valencia (VIU), 46002 Valencia, Spain

**Keywords:** out-of-school physical activity, diet, emotional attention, emotional clarity, emotional repair, young people, cluster analysis

## Abstract

There is an international social concern about the low levels of physical activity among young people. It is essential to know what factors influence the practice of physical activity in order to design effective proposals for health promotion. The study aims to: (1) classify primary school students according to their levels of out-of-school physical activity, Mediterranean diet, emotional attention, emotional clarity and emotional repair; (2) analyse descriptively and correlationally the adolescents’ profiles of out-of-school physical activity, Mediterranean diet and emotional attention, clarity and repair. The study design was cross-sectional and descriptive–correlational. The sample consisted of 293 children aged 10–12 years in Granada (Spain). An ad hoc socio-academic questionnaire, the KIDMED test and the Trait Meta-Mood Scale (TMMS-24) were used for data collection. Four clusters were identified by the Ward’s method and participants were classified using the K-means method. Subsequently, cluster classification was validated through the MANOVA test (*F* (861) = 106.12; *p* ≤ 0.001; *f* = 1.95). The strongest correlation was obtained in cluster 1 between emotional clarity and emotional repair (*r* = 0.56; *p* ≤ 0.01). In conclusion, the mean values of time spent in out-of-school physical activity, Mediterranean diet, emotional attention, emotional clarity and emotional repair of students are adequate and vary according to sex. There are significant differences among the physical activity levels of all clusters, as well as among the emotional variables of attention, clarity and repair. In addition, the correlations between the variables studied vary in each cluster.

## 1. Introduction

Several studies have highlighted the need to investigate physical, emotional and social development during this stage of human development [1,2], as it is during this period that behavioural habits are acquired that will be maintained during adulthood [3], such as the physical activity practice [4]. The study by Zych et al. [5] states that alcohol and tobacco consumption begins to originate in the early stages of adolescence, being related to increased social recognition. Another habit of special concern is physical activity, as more than 81% of schoolchildren do not comply with the daily physical practice recommendations [6], showing high levels of sedentary lifestyles [7]. Furthermore, physical activity levels tend to decrease with increasing age [8], as does diet quality [9].

Social concern about the healthy habits of young people stems partly from the existing scientific evidence, since, despite the proven health benefits of maintaining certain habits, the majority of recommendations are not complied with. In particular, it has been observed that physical activity has various physical, psychological and social health benefits [10,11], such as the prevention of cardiovascular diseases and increased body mass index, improvement of cardiometabolic health, and maintenance of physical fitness [12,13,14,15]. Several benefits of physical activity on an emotional level have also been demonstrated [16], although there are fewer studies on this in the scientific literature. It has been shown that, when subjected to negative emotional states, the physical activity practice helps the secretion of neurotransmitters that help to promote a sense of well-being in young people [17]. It has also been observed that through the practice of sport, the control of emotions and negative emotional states is promoted, which helps to educate young people emotionally [18]. Li et al. [16] found that young people who engage in higher levels of weekly physical activity show higher recognition in the dimensions that make up emotional intelligence.

Maintaining a healthy diet has health benefits for young people [19]. Despite this, positive adherence may be conditioned by elements such as the culture or socioeconomic status of the parents [20]. In particular, the Mediterranean area has a healthy dietary pattern known as the Mediterranean diet [21]. This pattern is characterised by the intake of fresh produce, fruit, vegetables, dairy products together with oily fish and a low consumption of red meat and products containing saturated fats [8]. Among the adolescent population, low adherence to this dietary pattern has been observed [22], opting for a low-calorie food intake that leads to poorer health [19]. In contrast, positive adherence to the Mediterranean diet has been shown to provide psychological, physiological, physical and emotional benefits in the adolescent population [22].

The current society demands increasingly emotionally competent people from early stages of development [23]. In view of this, emotional intelligence is becoming increasingly important in different educational stages [24]. Based on the model proposed by Salovey et al. [25], emotional intelligence should not be interpreted as a unidimensional variable but as composed of other sub-variables such as attention, clarity and emotional repair. Emotional attention is defined as the emotional capacity focused on paying attention to the emotional states that the person experiences at different moments in time [25]. Emotional clarity translates to the ability to understand whether an emotional state has a positive or negative effect on people’s health [25]. The development of these three skills aids the development of emotional competence, which will foster young people’s ability to be emotionally competent during future stages of human development [8]. Future adults will also gain numerous physical and mental health benefits if they engage in physical activity in conjunction with the development of emotional competence [8].

In light of the above, the study has the following objectives:

(1) To classify primary school students according to their levels of out-of-school physical activity, Mediterranean diet, emotional attention, emotional clarity and emotional repair.
(2) To analyse descriptively and correlationally the adolescents’ profiles of out-of-school physical activity, Mediterranean diet and emotional attention, clarity and repair.

This study is justified by the need to identify the levels of physical activity, Mediterranean diet and emotional intelligence of schoolchildren in Granda (Spain), as well as the correlations between these habits, as no similar previous studies have been found. This will allow us to determine whether it is necessary to design health promotion proposals that incorporate these healthy habits. Furthermore, clustering and analysis of correlations between variables will allow in-depth investigation of these habits in homogeneous groups, so that potential health promotion designs could be more precise, effective and specific for each cluster. These individualised designs could bring numerous benefits, such as reducing intervention time and resources, as well as being able to prioritise intervention clusters.

## 2. Materials and Methods

### 2.1. Design and Subjects

A non-experimental, cross-sectional, descriptive–correlational study was carried out on a sample of 293 primary schoolchildren living in Granada (Spain). Specifically, the students belong to the last two years of primary school. Likewise, the sample is quite homogeneous since 147 young people belong to the male sex (50.2%) and 146 to the female sex (49.8%). The ages of the participants were between 11 and 12 years old (11.45 ± 0.31).

In order to study the variables mentioned in this population, two inclusion criteria were defined. The first was that the schoolchildren had to be at the primary education stage and the second was that the students had to be in the last two years of the educational stage (fifth and sixth years of primary education).

Non-random sampling with accessibility criteria was applied, in which several schools offering primary education in Granada took part, willing to participate in the research unselfishly. The sample selected was representative, resulting in a sampling error of less than 0.05, for a confidence level of 0.95.

### 2.2. Instrument and Variables

An ad hoc questionnaire was used to obtain information on the sociodemographic and sports variables. This was used to collect the variables sex (male/female), age and time of weekly physical activity. For the collection of the latter variable, the following question was used: do you practice more than 3 h of out-of-school physical activity, with a dichotomous answer (yes/no). This question has already been used for other research [26]. A child who practices out-of-school physical activity for at least 3 h a week outside school is considered physically active, so this criterion was used, which is in line with international recommendations.

The Spanish version of the KIDMED questionnaire [27] was administered to measure Mediterranean diet. The questionnaire includes 16 dichotomous response items (yes/no). Each affirmative answer (yes) is valued with a positive point, with the exception of questions 5, 11, 13 and 15, for which an affirmative answer is assigned a negative point. The items answered negatively (no) are valued as zero points. Therefore, the final score ranges from −4 to 12 points. Based on the final score, the participant’s Mediterranean diet consumption can be classified as: optimal diet (≥8 points), needs improvement (2–7 points), or poor-quality diet (≤1). The reliability of this questionnaire in the present investigation was acceptable (α = 0.80).

The Trait Meta Mood Scale (TMMS-24) was also used. This scale was initially designed by Salovey et al. [25], although the Spanish version adapted by Fernández-Berrocal et al. [28] was administered. This version is made up of 24 items that are answered using a five-point Likert scale (from 1 = “disagree” to 5 = “completely agree”). The first eight items are intended to assess emotional attention. The second set of eight items assess emotional clarity. Finally, the last eight items assess emotional repair. Likewise, Cronbach’s alpha has evidenced the following values: emotional attention (α = 0.879), emotional clarity (α = 0.824) and emotional repair (α = 0.801). The categories of each emotional intelligence variable vary according to the score obtained and sex:

− Categories of emotional attention: needs improvement (score: male ≤ 21; female ≤ 24); appropriate (score: male 22–32; female 25–35) and excellent (score: male ≥ 33; female ≥ 36).
− Categories of emotional clarity: needs improvement (score: male ≤ 25; female ≤ 23); appropriate (score: male 26–35; female 24–34) and excellent (score: male ≥ 36; female ≥ 35).
− Categories of emotional repair: needs improvement (score: male ≤ 23; female ≤ 23); appropriate (score: male 24–35; female 24–34) and excellent (score: male ≥ 36; female ≥ 35).

### 2.3. Procedure

Before starting data collection, a letter was sent to the different schools in the city of Granada (Spain). Only two schools agreed to participate. In turn, the schools contacted the legal guardians of the young people. Parents were informed of the research, as they were notified of the objectives. They were also assured by the research team that the data would be treated anonymously and for scientific purposes. Once permission was obtained, the research team gained access to the centre. Likewise, the data were collected digitally, and the researchers were present during the response time to resolve any unforeseen issues that might arise. With respect to ethical criteria, the research followed the standards set out in the Declaration of Helsinki and was approved and supervised by an ethics committee from the University of Granada (2966/CEIH/2022).

### 2.4. Data Analysis

The statistical analysis was carried out with IBM SPP 26.0 software (International Business Machines Corporation, Armonk, NY, USA). Three phases were followed: a first phase of descriptive and correlational statistics of the entire sample, a second phase of creation and corroboration of clusters, and a third phase of descriptive-correlational statistics of each cluster.

At the beginning of the first phase, the data were cleaned. Afterwards, a descriptive statistical analysis of the variables according to the sex of the participants was performed. Chi-square and Cramer’s V tests were used to identify if there were differences in the values according to sex. Subsequently, the data of the variables were typified (z-scores) in order to study their correlations. No values were eliminated since no outliers were obtained (greater than 3.0 or less than −3.0) that could have distorted the subsequent cluster analysis [29]. The Kolmogorov-Smirnov test was applied to determine the type of distribution and the Spearman correlation test was applied when non-normal distributions of the variables were obtained (*p* ≤ 0.05). The t-Welch test was applied to compare the mean values of the variables according to sex.

In the second phase of the statistical analysis, participants were classified according to their levels of out-of-school physical activity, Mediterranean diet, emotional attention, emotional clarity, and emotional repair according to the process established by Hair et al. [29]. First, Ward’s agglomerative hierarchical procedure was applied, using the squared Euclidean distance to identify the optimal number of clusters. This procedure reduces the distance between subjects in each cluster and avoids creating long chains [30]. Subsequently, the k-means classification method was applied to identify the subjects in each group based on the groupings from the previous process. In social research it is common to use Ward’s method and k-means together to perform clustering of participants [31]. Second, the MANOVA test was used to validate the clustering. For this purpose, cluster membership was used as an independent variable and out-of-school physical activity, Mediterranean diet, emotional attention, emotional clarity and emotional repair were used as dependent variables. The effect between variables was identified by applying Pillai’s trace test [32] and the equality of error variances with Levene’s test. Afterwards, the Games-Howell post hoc was applied because the Levene test obtained values of *p* > 0.05.

In the third phase of the statistical study, a descriptive–correlational analysis of the research variables was performed according to each cluster. Chi-square and Cramer’s V tests were applied to identify differences according to the sex of the participants. In addition, correlations between variables were calculated with Spearman’s test.

## 3. Results

Table 1 shows the descriptive statistics for out-of-school physical activity, Mediterranean diet, attention, emotional clarity and emotional repair according to the sex of the participants. The majority of schoolchildren perform more than 300 min of out-of-school physical activity per week, have a Mediterranean diet of average quality and have adequate emotional attention, emotional clarity and emotional repair. In addition, no significant differences were obtained between the categories of the study variables according to the sex of the children.

For the participants as a whole, significant correlations were found between physical activity and Mediterranean diet (r = −0.13, *p* ≤ 0.05), Mediterranean diet and emotional clarity (r = −0.19, *p* ≤ 0.01), Mediterranean diet and emotional repair (r = −0.14, *p* ≤ 0.05), emotional attention and emotional clarity (r = 0.33, *p* ≤ 0.01), emotional attention and emotional repair (r = 0.34, *p* ≤ 0.01) and between emotional clarity and emotional repair (r = 0.45, *p* ≤ 0.01). In addition, Table 2 presents the mean descriptive statistics z-scores of the variables and their correlations according to the sex of the participants. Regardless of sex, positive and significant correlations were obtained between emotional attention and emotional clarity, between emotional attention and emotional repair, and between emotional clarity and emotional repair.

There is no significant difference between the levels of out-of-school physical activity (z-scores) of males and females (t-Welch = 2.86; *p* = 0.09). Similarly, there are no significant differences in terms of Mediterranean diet (t-Welch = 0.07; *p* = 0.80), emotional attention (t-Welch = 0.01; *p* = 0.94), emotional clarity (t-Welch = 0.41; *p* = 0.52) and emotional repair (t-Welch = 0.52; *p* = 0.47).

It was decided to select four clusters based on the results of the squared Euclidean distance proximity matrix, the clustering history and the dendrogram obtained from Ward’s agglomerative hierarchical procedure. Table 3 shows the descriptive statistics of the four clusters according to the k-means method classification.

The classification of the four clusters was validated with the MANOVA test, since the Pillai’s Trace values were: F (861) = 106.12; *p* ≤ 0.001; f = 1.95. In addition, the results of Levene’s test were significant for all research variables (physical activity: F(3, 87.17) = 826.08; *p* < 0.001; Mediterranean diet: F(3, 51.95) = 110.29; *p* < 0. 001; emotional attention: F(3, 28.33) = 39.56; *p* < 0.001); emotional clarity: F(3, 28.62) = 40.12; *p* < 0.001; emotional repair: F(3, 36.18) = 56.99; *p* < 0.001), so the Games–Howell post hoc test was applied. Table A1 shows the post hoc multiple comparisons among the four clusters according to the five research variables.

Table 4 shows the descriptive statistics of the research variables according to the sex of the adolescents and the cluster to which they belong.

In clusters 1, 3 and 4, there were no significant differences according to sex for any of the research variables. Regarding cluster 2, significant differences were found in the practice of physical activity (x^2^ (1) = 4.52, *p* = 0.03; V = 0.26) according to sex.

Table 5 presents the descriptive z-scores and Spearman correlation statistics of the variables for each cluster. The values of the five classification variables for each of the four clusters follow normal distributions according to the Kolmogorov-Smirnov test (*p* ≤ 0.001). In the first cluster, two positive and moderate significant correlations (0.40 < r < 0.60) were found between emotional attention and emotional clarity (r = 0.41, *p* ≤ 0.05) and between emotional clarity and emotional repair (r = 0.56, *p* ≤ 0.01). In the third cluster, two significant negative and weak correlations (0.20 < r < 0.40) were obtained between Mediterranean diet and emotional clarity (r = −0.25, *p* ≤ 0.05) and between emotional attention and emotional repair (r = −0.31, *p* ≤ 0.05).

Based on the results in Table 3, Table 4 and Table 5, it can be observed that the first cluster is the least numerous, representing 12.97% of young people, and has the highest proportion of females (57.89%). It is the group with the worst practice of out-of-school physical activity, as none of its members exceeds 300 min/week. It is the second group with the best Mediterranean diet, emotional attention and emotional clarity. Only a positive, moderate and significant correlation was found between attention and emotional clarity and between emotional clarity and emotional repair.

The second cluster includes 22.53% of the students. It is the group with the second worst average physical activity, although 93.94% of its members do more than 300 min/week of out-of-school physical activity, and significant differences were obtained in favour of males according to gender. Moreover, it is the group with the second worst Mediterranean diet, although there are no participants with a low-quality diet. On an emotional level, it is the group with the highest mean scores in the three variables studied. It should be noted that physical activity is negatively related to all the other variables, although not significantly so. The Mediterranean diet is also negatively associated with the three emotional variables, although it is significantly and slightly associated with emotional attention and emotional clarity.

The third group is made up of 18.77% of the participants. This group and the fourth have the best mean score for out-of-school physical activity. It is the group with the worst Mediterranean diet, although all individuals have a high-quality diet. Most of the students obtained adequate values for the emotional variables. Although there are no significant relationships, emotional attention is negatively linked to emotional clarity and emotional repair.

The majority of young people are in the fourth cluster (45.73%). The mean scores for physical activity and diet are the highest of all the groups, but these are the worst for the emotional variables. In this group, no significant relationships were found between the variables, although it should be noted that most of them were positive.

## 4. Discussion

The aims of the study were: (1) to classify primary school students according to their levels of out-of-school physical activity, Mediterranean diet, emotional attention, emotional clarity and emotional repair; (2) to analyse descriptively and correlationally the adolescents’ profiles of out-of-school physical activity, Mediterranean diet and emotional attention, clarity and repair.

The number of previous studies that have analysed the relationship between emotional intelligence and healthy habits is very limited [33], and even fewer studies exist involving young people [34]. Therefore, due to the novelty of this research, there is some difficulty in discussing the results found on the basis of scientific evidence.

Ward’s method made it possible to identify four classification groups of students. In turn, the k-means method made it possible to classify each participant into one of the four groups according to their levels of the study variables. This classification has been validated through the MANOVA test [35]. Furthermore, with the application of both Cluster methods, it has been possible to form groups that are as internally homogeneous as possible and as heterogeneous as possible among them [31], also obtaining significant differences between the mean values of the variables in a cluster with respect to those of the other groups. This means that we have obtained a classification of good discriminative statistical quality.

The profiles of the clusters are different, even more so when looking at the results for the student body as a whole. The majority of adolescents engage in more than 300 min of out-of-school physical activity, have an average quality Mediterranean diet and adequate attention, clarity and emotional repair.

It is noteworthy that 85.67% of schoolchildren in Granada engage in more than 300 min of out-of-school physical activity per week. This is in line with other studies in which most participants were physically active [36,37] and in contrast to many others, such as that by Guthold et al. [6] who found that over 81% of young people worldwide were inactive. In addition, the trend of females having lower levels of physical activity than males continues [38,39].

The average quality of dietary patterns of young people in Granada is higher than that shown in other studies [22,40,41] and lower than those found by García, Carrillo and Guillamon [42], who showed that 24% of primary school students had a low-quality diet and 46.6% had a high-quality diet (Granada students had 31.06% and 31.74%, respectively). It would be advisable to design a proposal to promote a healthy diet, since, although the results obtained are not bad, they tend to worsen after the age of 11 because young people begin to take control of their own diet [43,44].

The average levels of emotional attention, emotional clarity and emotional repair of primary school pupils in Granada are mostly adequate. Despite the above, of the three emotional variables, emotional attention is the one that obtains the lowest levels. Jauk and Ehrenthal [45] and Núñez and Muñoz [46] also demonstrated that primary school students showed adequate emotional control. Dale et al. [47] concluded their systematic review by finding that physical activity reduces depression and its symptoms, while improving physical self-perception. Perhaps, emotional intelligence could be the moderating variable conditioning the relationship between physical activity and the psychological benefits it brings, and further research is needed.

Zysberg and Hemmel [48], Shuk-Fong and Hsiu-Hua [49] and Omar et al. [50] found that physical activity was positively related to emotional intelligence in adults. This is in line with the findings for male students in Granada, but not for female students, as the relationship was negative, although not significant. Perhaps the discrepancy between the results of these studies and the one conducted in Granada is not only due to the age of the participants, but also to the fact that in the adult studies emotional intelligence was measured as the only variable and in the one conducted in Granada the relationships with the three attributes of emotional intelligence were studied separately (attention, clarity and repair). No similar studies with children and adolescents have been identified.

The physical activity of students in Granada is negatively related to their diet habits. This is equally true for both males and females. Moreover, there is also a negative relationship between the variables in the second cluster, which is the only one in which the correlation test could be applied. These results differ from those found in other research, where positive relationships were found between diet and physical activity [51,52,53]. Perhaps, emotional intelligence could be the variable that conditions the relationship between physical activity and diet, as demonstrated by Melguizo-Ibánez et al. [8].

Because of the above, and especially because the levels of the variables studied and their correlations vary according to different factors and groupings, it is recommended that detailed cluster studies be carried out so that more effective health promotion proposals can be designed [54].

The study conducted has some limitations. Firstly, there is a limitation associated with the type of cross-sectional study, as it is based on a single measurement at an exact moment in time. This implies that the measurement values obtained may have varied over time and that causal relationships between the study variables cannot be established. Secondly, there is a limitation in the measurement of physical activity, because a subjective instrument was used, so it is not as accurate as the accelerometer could be. Despite this limitation, physical activity questionnaires are the most practical and frequently used instruments in epidemiological studies [55], because they allow information to be obtained from a large number of people in a short time and at an efficient cost [56]. As a result, it was possible to obtain a representative sample of students in the city of Granada (Spain).

Regarding future lines of research, it would be interesting to carry out further studies on the other habits of primary school students to identify whether the number of classification clusters is maintained independently of the study factors. If the number of clusters were to remain at four, perhaps a new area of study could be opened up in the scientific field. It would also be relevant to know how the dimensions of emotional intelligence influence other healthy habits, such as smoking and alcohol consumption. Similarly, a longitudinal study could be designed involving young people, adults and older people from different countries, to identify how their levels of physical activity, diet and emotional intelligence vary with age and how they vary over time, which would allow causal relationships to be established. It would also be appropriate to design school health promotion interventions. These proposals could be designed by educational professionals including people from different age groups, with the aim of improving the quality of life of future generations and based on the principle ‘the example comes from the top’ [57].

## 5. Conclusions

The mean values of time spent in out-of-school physical activity, Mediterranean diet, emotional attention, emotional clarity and emotional repair of primary school students in Granada (Spain) are adequate, although they could be improved, and vary according to sex. Similarly, the relationships between the variables also vary according to sex.

Four clusters of primary school students were obtained according to the study variables. There are significant differences among the physical activity levels of all clusters, as well as among the emotional variables of attention, clarity and repair. The majority of students belong to cluster 4, which is characterised by having the highest levels of physical activity and diet, the lowest levels of emotional attention, emotional clarity and emotional repair and non-significant relationships among all variables, these being predominantly positive.

The individual analysis of each cluster has allowed the identification of priority and effective lines of intervention. For example, although it would be advisable to design proposals to promote healthy habits and emotional intelligence for all schoolchildren, priority should be given to cluster 1 over the rest, especially physical activity, but without forgetting diet and emotional intelligence.

## Figures and Tables

**Table 1:** Descriptive statistics of the research variables according to sex.

Variable	Categories	N	Sex N (%)		Chi-Square (*p*-Value)
			Male	Female	
Out-of-school physical activity	Less than 300 min/week	42	16 (38.10)	26 (61.90)	2.86 (*p* = 0.09)
	More than 300 min/week	251	131 (52.19)	120 (47.81)	
Mediterranean diet	Poor quality	34	20 (58.82)	14 (41.18)	3.11 (*p* = 0.21)
	Medium quality	166	76 (42.17)	90 (57.83)	
	High quality	93	51 (54.84)	42 (45.16)	
Emotional attention	Needs improvement	91	43 (47.25)	48 (52.75)	2.06 (*p* = 0.36)
	Appropriate	162	87 (53.70)	75 (46.30)	
	Excellent	40	17 (42.50)	23 (57.50)	
Emotional clarity	Needs improvement	81	38 (46.91)	43 (53.09)	0.49 (*p* = 0.78)
	Appropriate	166	85 (51.20)	81 (48.80)	
	Excellent	46	24 (52.17)	22 (47.83)	
Emotional repair	Needs improvement	56	30 (53.57)	26 (46.43)	0.52 (*p* = 0.77)
	Appropriate	169	85 (50.30)	84 (49.70)	
	Excellent	68	32 (47.06)	36 (52.94)	

**Table 2:** Descriptive statistics and z-score correlations of the research variables according to sex.

		M (SD)	CI 95%	K-S	Skewness	Kurtosis	2	3	4	5
Male	1. PA	0.10 (0.89)	−0.05/0.24	<0.001	−2.54	4.50	−0.01	0.11	0.22 **	0.14
	2. MD	−0.02 (1.06)	−0.19/0.16	<0.001	0.26	−0.76	-	−0.14	−0.24 **	−0.15
	3. EA	0.00 (0.95)	−0.16/0.00	<0.001	0.13	−0.46	-	-	0.22 **	0.25 **
	4. EC	0.04 (0.99)	−0.12/0.20	<0.001	0.09	−0.58	-	-	-	0.38 **
	5. ER	−0.04 (1.00)	−0.21/0.12	<0.001	−0.01	−0.61	-	-	-	-
Female	1. PA	−0.10 (1.09)	0.00/0.41	<0.001	−1.70	0.90	−0.24 **	−0.09	−0.16	−0.03
	2. MD	0.02 (0.94)	−0.14/0.17	<0.001	0.07	−0.33	-	−0.01	−0.13	−0.13
	3. EA	0.00 (1.05)	−0.17/0.18	<0.001	0.22	−0.82	-	-	0.44 **	0.42 **
	4. EC	−0.04 (1.01)	−0.20/0.13	<0.001	0.16	−0.67	-	-	-	0.53 **
	5. ER	0.04 (1.00)	−0.12/0.21	<0.001	−0.07	−0.61	-	-	-	-

Note. Physical Activity (PA); Mediterranean diet (MD); Emotional attention (EA); Emotional clarity (EC); Emotional repair (ER); Mean (M); Standard deviation (SD); Confidence interval (CI); Kolmogorov-Smirnov (K-S); ** *p* ≤ 0.01.

**Table 3:** Descriptive statistics for the four k-means clusters.

	N	Sex (N)		Cluster Centre (z-Cores)				
		Male	Female	PA M (SD)	MD M (SD)	EA M (SD)	EC M (SD)	ER M (SD)
Cluster 1	38	16	22	−2.44 (0.00)	0.36 (1.08)	−0.18 (0.87)	−0.22 (0.93)	−0.27 (0.96)
Cluster 2	66	34	32	0.24 (0.68)	−0.40 (0.80)	1.00 (0.91)	1.00 (0.78)	1.13 (0.65)
Cluster 3	55	28	27	0.41 (0.00)	−1.27 (0.00)	−0.29 (0.81)	−0.26 (0.82)	−0.26 (0.79)
Cluster 4	134	69	65	0.41 (0.00)	0.62 (0.62)	−0.32 (0.82)	−0.32 (0.86)	−0.37 (0.82)

Note. Physical Activity (PA); Mediterranean diet (MD); Emotional attention (EA); Emotional clarity (EC); Emotional repair (ER); Mean (M); Standard deviation (SD).

**Table 4:** Descriptive statistics for each cluster according to the sex of the participants.

Cluster	Variable	Categories	N	Sex (N)		Chi-Square (*p*-Value)
				Male	Female	
Cluster 1	Out-of-school physical activity	Less than 300 min/week	38	16	22	NA
		More than 300 min/week	0	0	0	
	Mediterranean diet	Poor quality	9	3	6	4.50 (*p* = 0.09)
		Medium quality	21	7	14	
		High quality	8	6	2	
	Emotional attention	Needs improvement	13	6	7	1.56 (*p* = 0.46)
		Appropriate	23	10	13	
		Excellent	2	0	2	
	Emotional clarity	Needs improvement	13	8	5	4.49 (*p* = 0.11)
		Appropriate	22	8	14	
		Excellent	3	0	3	
	Emotional repair	Needs improvement	10	5	5	1.28 (*p* = 0.53)
		Appropriate	23	10	13	
		Excellent	5	1	4	
Cluster 2	Out-of-school physical activity	Less than 300 min/week	4	0	4	4.52 (*p* = 0.03)
		More than 300 min/week	62	34	28	
	Mediterranean diet	Poor quality	0	0	0	0.58 (*p* = 0.45)
		Medium quality	36	17	19	
		High quality	30	17	13	
	Emotional attention	Needs improvement	3	2	1	1.61 (*p* = 0.45)
		Appropriate	29	17	12	
		Excellent	34	15	19	
	Emotional clarity	Needs improvement	0	0	0	0.00 (*p* = 0.99)
		Appropriate	31	16	15	
		Excellent	35	18	17	
	Emotional repair	Needs improvement	0	0	0	1.78 (*p* = 0.18)
		Appropriate	15	10	5	
		Excellent	51	24	27	
Cluster 3	Out-of-school physical activity	Less than 300 min/week	0	0	0	NA
		More than 300 min/week	55	28	27	
	Mediterranean diet	Poor quality	0	0	0	NA
		Medium quality	0	0	0	
		High quality	55	28	27	
	Emotional attention	Needs improvement	21	8	13	2.93 (*p* = 0.23)
		Appropriate	33	19	14	
		Excellent	1	1	0	
	Emotional clarity	Needs improvement	18	6	12	3.38 (*p* = 0.18)
		Appropriate	35	21	14	
		Excellent	2	1	1	
	Emotional repair	Needs improvement	11	6	5	0.07 (*p* = 0.96)
		Appropriate	40	20	20	
		Excellent	4	2	2	
Cluster 4	Out-of-school physical activity	Less than 300 min/week	0	0	0	NA
		More than 300 min/week	134	69	65	
	Mediterranean diet	Poor quality	25	17	8	3.35 (*p* = 0.07)
		Medium quality	109	52	57	
		High quality	0	0	0	
	Emotional attention	Needs improvement	54	27	27	0.54 (*p* = 0.76)
		Appropriate	77	41	36	
		Excellent	3	1	2	
	Emotional clarity	Needs improvement	50	24	26	2.68 (*p* = 0.26)
		Appropriate	78	40	38	
		Excellent	6	5	1	
	Emotional repair	Needs improvement	35	19	16	0.65 (*p* = 0.72)
		Appropriate	91	45	46	
		Excellent	8	5	3	

Note. Not applicable (NA).

**Table 5:** Descriptive statistics (z-scores) and correlations between variables in each cluster.

		K-S	Skewness	Kurtosis	2	3	4	5
Cluster 1	PA	NA	NA	NA	NA	NA	NA	NA
	MD	0.28 ***	−0.03	−0.70	-	−0.05	−0.05	0.01
	EA	0.35 ***	0.03	−0.45	-	-	0.41 *	0.20
	EC	0.33 ***	0.16	−0.44	-	-	-	0.56 **
	ER	0.03 ***	0.09	−0.32	-	-	-	-
Cluster 2	PA	0.54 ***	−3.78	12.59	−0.10	−0.23	−0.24	−0.14
	MD	0.36 ***	−0.19	−2.03	-	−0.27 *	−0.25 *	−0.06
	EA	0.33 ***	−0.58	−0.58	-	-	0.08	−0.31 *
	EC	0.36 ***	−0.12	−2.05	-	-	-	0.00
	ER	0.48 ***	−1.33	−0.23	-	-	-	-
Cluster 3	PA	NA	NA	NA	NA	NA	NA	NA
	MD	NA	NA	NA	-	NA	NA	NA
	EA	0.38 ***	−0.18	−1.09	-	-	−0.12	−0.11
	EC	0.38 ***	−0.16	−0.52	-	-	-	0.13
	ER	0.40 ***	−0.22	0.72	-	-	-	-
Cluster 4	PA	NA	NA	NA	NA	NA	NA	NA
	MD	0.50 ***	1.63	0.66	-	0.02	−0.06	0.11
	EA	0.36 ***	−0.04	−1.05	-	-	0.01	0.13
	EC	0.35 ***	0.07	−0.67	-	-	-	0.13
	ER	0.39 ***	−0.16	−0.01	-	-	-	-

Note. Physical Activity (PA); Mediterranean diet (MD); Emotional attention (EA); Emotional clarity (EC); Emotional repair (ER); Kolmogorov-Smirnov (K-S); *** *p* ≤ 0.001; ** *p* ≤ 0.01; * *p* ≤ 0.05; not applicable (NA).

## Data Availability

Not applicable.

## Appendix A

**Table A1:** Post hoc multiple comparisons of variables in clusters.

	Cluster (I)	Cluster (J)	Mean Difference (I − J)	Error Deviation	Sig.	Confidence Interval (95%)	
						Lower Limit	Lower Limit
PA	1	2	−2.68	0.08	*	−2.90	−2.45
	1	3	−2.85	0.00	NA	−2.85	−2.85
	1	4	−2.85	0.00	NA	−2.85	−2.85
	2	3	−0.17	0.08	0.181	−0.40	0.05
	2	4	−0.17	0.08	0.181	−0.39	0.05
	3	4	0.00	0.00	NA	0.00	0.00
MD	1	2	0.77	0.20	0.002	0.24	1.30
	1	3	1.63	0.17	*	1.16	2.11
	1	4	−0.26	0.18	0.510	−0.74	0.23
	2	3	0.87	0.10	*	0.61	1.13
	2	4	−1.02	0.11	*	−1.31	−0.73
	3	4	−1.89	0.05	*	−2.03	−1.75
EA	1	2	−1.17	0.18	*	−1.65	−0.70
	1	3	0.11	0.18	0.918	−0.35	0.58
	1	4	0.14	0.16	0.811	−0.28	0.56
	2	3	1.29	0.16	*	0.88	1.69
	2	4	1.31	0.13	*	0.97	1.66
	3	4	0.02	0.13	0.997	−0.31	0.37
EC	1	2	−1.22	0.18	*	−1.69	−0.75
	1	3	0.04	0.19	0.996	−0.45	0.53
	1	4	0.10	0.17	0.932	−0.34	0.54
	2	3	1.27	0.15	*	0.88	1.65
	2	4	1.33	0.12	*	1.01	1.64
	3	4	0.06	0.12	0.973	−0.29	0.41
ER	1	2	−1.39	0.17	*	−1.85	−0.93
	1	3	−0.01	0.19	1.000	−0.50	0.49
	1	4	0.11	0.17	0.922	−0.34	0.56
	2	3	1.38	0.13	*	1.04	1.73
	2	4	1.50	0.11	*	1.22	1.77
	3	4	0.11	0.13	0.807	−0.22	0.45

Note. Physical Activity (PA); Mediterranean diet (MD); Emotional attention (EA); Emotional clarity (EC); Emotional repair (ER); Mean (M); Standard deviation (SD); Confidence interval (CI); * *p* ≤ 0.001; not applicable (NA).
